# Effects of Soy Protein on Liver and Adipose Tissue Inflammation and Gut Microbiota in Mice Fed with Ketogenic Diets

**DOI:** 10.3390/nu17152428

**Published:** 2025-07-25

**Authors:** Wen-Keng Li, I-Ting Wu, Wan-Ju Yeh, Wen-Chih Huang, Hsin-Yi Yang

**Affiliations:** 1 Department of Nutritional Science, Fu Jen Catholic University, New Taipei City 242, Taiwan; 2 Graduate Program of Nutrition Science, National Taiwan Normal University, Taipei 116, Taiwan; wandayeh@ntnu.edu.tw; 3 Department of Anatomical Pathology, Far Eastern Memorial Hospital, New Taipei City 220, Taiwan

**Keywords:** soy protein, fatty liver, ketogenic diet, inflammation, gut microbiota

## Abstract

**Background**: Studies on ketogenic diets with a higher percentage of fat composition have revealed conflicting results regarding the modulation of lipid metabolism and tissue inflammation. Furthermore, studies on soy protein consumption in ketogenic diets remain limited. In this study, the effects of ketogenic diets on hepatic and adipose tissue inflammation and of soy protein replacement in ketogenic diets were investigated. **Methods**: Mice were randomly assigned to a control diet (C), ketogenic diet (KD), or ketogenic with soy protein (KS) groups for an 18-week experiment. Both ketogenic diet groups were fed a low-carbohydrate, high-fat diet during the first 12 weeks and a ketogenic diet during the last 6 weeks of the experiment. The KS group was fed the same diet as the KD group, but soy protein was substituted for casein during the last 6 weeks. **Results**: The KD and KS groups exhibited higher plasma β-hydroxybutyrate levels; a higher incidence of hyperlipidemia; and lower blood glucose, mesenteric fat mass, adipose tissue TNF-α, IL-1β levels, and NLRP3 protein expression compared with the C group. In the gut microbiota analysis, the KD group had a higher F-B ratio than the C group. Greater *A. muciniphila* abundance and a lower F-B ratio were noted in the KS group compared with the KD group. **Conclusions**: Although ketogenic diets decreased mesenteric fat mass and adipose tissue inflammation and modulated NLRP3 expression, they were associated with hepatic inflammation and gut dysbiosis. Soy protein consumption in a ketogenic diet did not differ from casein consumption regarding diet-induced tissue inflammation, but it may have altered the gut microbiota.

## 1. Introduction

The prevalence of overweight and obesity has increased over time, primarily due to changes in dietary habits and lifestyle. Obesity has been linked to numerous chronic diseases, such as fatty liver disease, cardiovascular disease, and type II diabetes [1]. Therefore, weight loss strategies have become a major focus worldwide. In addition to medical intervention or surgery for patients with severe obesity, lifestyle modification, including increased physical activity and improved dietary habits, plays a crucial role in obesity treatment [2].

Although energy restriction and low-fat diets have conventionally been recommended, the effectiveness of long-term weight control is complex, and adherence remains challenging. The ketogenic diet, characterized by a very low percentage of carbohydrates as a total energy source and the use of ketone bodies derived from fatty acid oxidation as an alternative energy source, has attracted increasing attention for its effectiveness in rapid weight reduction [3]. However, the benefits and drawbacks of ketogenic diets remain inconclusive. In a rodent model, the ketogenic diet decreased weight and inflammatory responses in white adipose tissue (WAT) but caused hepatic fat accumulation and inflammation [4]. The inconsistent results of the ketogenic diets have highlighted concerns regarding their possible side effects and long-term consequences, such as dyslipidemia and systemic inflammation [5]. Additionally, rigorous limitation of carbohydrate intake accompanied by high fat and protein consumption may disrupt the distribution of gut microbiota [6], damage intestinal tight junctions, increase gut leakiness and hyperendotoxemia, thereby inducing abnormal metabolic and inflammatory responses in other tissues [7].

With the increasing prevalence of vegetarianism, the consumption of soy protein and vegetable oils to meet dietary needs merits further exploration. Lipids comprise at least 65–70% of total energy sources in ketogenic diets. Animal-based diets generally contain more saturated fatty acids and cholesterol than plant-based diets, which can increase the risk of cardiovascular disease and chronic inflammation [8]. Soy is a major source of dietary protein for vegetarians. In addition to being a high-quality protein source, soy features compositional characteristics potentially associated with key bioactive functions, such as antioxidative, anti-inflammatory, and lipid-lowering effects [9]. Recent studies have also indicated that soy protein may provide sufficient nitrogen and energy to maintain gut microbiota growth [10] and regulate lipid metabolism through gut microbiota alteration [11].

Although the ketogenic diet has gained heightened attention for its potential to facilitate rapid weight loss, existing research findings are inconsistent regarding the effect of this extreme dietary practice on metabolism and inflammatory responses. For example, the effect of the ketogenic diet on appetite may underlie its weight loss effectiveness [12]. In addition to the limited evidence regarding whether this extremely high fat consumption associated with the ketogenic diet affects metabolism through the gut–liver axis, the health effects of different protein sources in the ketogenic diets require further clarification. Accordingly, we examined whether the transition from a low-carbohydrate, high-fat diet to a ketogenic diet affected intestinal health and liver inflammation in a mouse model. Additionally, we explored the potential effects of different dietary proteins and their related mechanisms. Mice were also pair-fed with isocaloric diets to ensure they consumed the same amount of energy and protein, preventing the influence of different appetites on our results.

## 2. Materials and Methods

### 2.1. Experimental Design

Twenty-seven 5-week-old male C57BL/6 mice were purchased from the National Laboratory Animal Breeding and Research Center (Taipei, Taiwan). All study protocols were reviewed and approved by the Institutional Animal Care and Use Committee of Taipei Medical University (LAC-2019-0050). Environmental conditions for the mice were maintained in a room at 22 ± 2 °C, 55% ± 5% humidity and a 12 h light/dark cycle. To ensure group comparability and maintain animal welfare, mice were housed three per cage, with stable cage compositions throughout the study to avoid aggression in adult C57BL/6 mice [13]. All mice were weighed individually before grouping, and the average body weight per cage was calculated. Cages were then randomly assigned to one of three experimental groups using a random number generator sequence, helping balance the distribution of initial cage weights across groups. Mice were randomly assigned to control (C), ketogenic diet (KD), or ketogenic diet with soy protein (KS) groups (n = 9/group) for an 18-week experiment after a 1-week liquid-diet adaptation period. For the first 12 weeks, group C was fed a control liquid diet (total energy: 79% carbohydrates, 9% lipids, and 12% protein), and the other two groups were fed a low-carbohydrate, high-fat liquid diet (total energy: 18% carbohydrates, 70% lipids, and 12% protein) for the mice to become accustomed to the high-fat liquid diet intake. For the following 6 weeks, group C was fed the same control liquid diet and groups KD and KS were fed a ketogenic diet containing more lipids and fewer carbohydrates (total energy: 5% carbohydrates, 83% lipids, and 12% protein) and different protein sources (casein for KD and soy protein for KS), in alignment with a previously reported method [14] (Supplementary Table S1). During the experimental period, food intake was measured daily, and mice were pair-fed with isocaloric diets to ensure they consumed the same amount of energy and protein. Body weights were recorded weekly. At the end of the experiment, fresh fecal samples were collected, after which mice were anesthetized with isoflurane and euthanized. Blood, liver, and abdominal adipose tissue were collected for further analysis.

### 2.2. Blood Analysis

Blood samples were collected from the inferior vena cava, and blood glucose and ketone body levels were measured with a blood-glucose-ketone meter (ApexBio AB-302G, Hsinchu, Taipei). Plasma samples were collected after blood samples were mixed with heparin and centrifuged, and stored at −80 °C until analysis. Plasma triglycerides (TGs), total cholesterol (TC), high-density lipoprotein-cholesterol (HDL-C), low-density lipoprotein-cholesterol (LDL-C), alanine aminotransferase (ALT), and aspartate aminotransferase (AST) were analyzed with a Roche Modular P800 autoanalyzer (Roche Diagnostics, Indianapolis, IN, USA). Free fatty acid (FFA) concentrations were detected with commercial kits (BioVision, K612-100, Milpitas, CA, USA). Endotoxin levels were determined according to the limulus amebocyte lysate method (L00350, GenScript, Piscataway, NJ, USA). Insulin, fibroblast growth factor 21 precursor (FGF21), leptin, and adiponectin levels were measured through enzyme-linked immunosorbent assays (ELISA) with commercial kits (Mercodia Mouse Insulin ELISA 10-1247-01. Uppsala, Sweden; Mouse and Rat FGF21 ELISA, Quantikine MF2100, Minneapolis, MN, USA; Mouse and Rat Leptin ELISA, Invitrogen, Carlsbad, CA, USA; AssayMax Mouse Adiponectin ELISA, Assaypro EMA2500-1, St. Charles, MO, USA). Malondialdehyde (MDA) levels were measured spectrophotometrically through a thiobarbituric acid reactive substances assay [15].

### 2.3. Liver Sample Analysis

Liver samples were collected and weighed after perfusion with ice-cold saline. The samples were then fixed in 10% (*v*/*v*) formaldehyde and then stained with hematoxylin and eosin for histopathological analysis. Morphological changes were examined by a pathologist blinded to group assignments. For hepatic lipid analysis, samples were homogenized and extracted with chloroform/methanol [16], and triglycerides and cholesterol concentrations were measured with commercial kits (Randox TR210 and fortress BXC0271, Antrim, UK). Liver tissues were homogenized with a buffer (50 mM Tris-HCl, 150 mM NaCl, 1% NP-40, 0.1% sodium dodecyl sulfate [SDS]) containing a protease inhibitor (Roche, Mannheim, Germany); after centrifugation, the supernatant was collected for further analysis. FFA and MDA concentrations were analyzed according to the procedure described in 2.2. Levels of the proinflammatory cytokines tumor necrosis factor (TNF) α and interleukin (IL) 1β were measured with ELISA commercial kits (Mouse TNF-α DuoSet ELISA, DY-410-05; Mouse IL-1β/IL-1F2 DuoSet ELISA, DY-401-05, R&D Systems, Minneapolis, MN, USA). The protein concentration of the homogenates was measured with a Bio-Rad protein assay dye (Bio-Rad Laboratories, Hercules, 500-0002, CA, USA).

### 2.4. Adipose Tissue Analysis

Abdominal WAT, including epididymal, perirenal, and mesenteric adipose tissue, was collected and weighed. MDA, TNF-α, and IL-1β concentrations were analyzed as described in Section 2.3.

### 2.5. Western Blotting

The inflammation-related protein expression of liver and adipose tissue was analyzed through Western blotting. Samples were homogenized with a 50 mM Tris-HCl, 50 mM NaCl, 1% NP-40, and 0.1% SDS solution containing protease inhibitors. After centrifugation, the supernatant was collected. Homogenate containing 30 μg protein was separated on an SDS-polyacrylamide gel and transferred to a polyvinylidene difluoride membrane. Nonspecific binding sites were blocked through membrane incubation overnight at 4 °C in non-fat milk. The membranes were than washed with phosphate-buffered saline/Tween-20 and incubated with an anti-toll-like receptor 4 (TLR4) antibody (monoclonal antibody to TLR4, IMG-5031A, Novus Biologicals, Centennial, CO, USA), anti-myeloid differentiation primary response 88 (MyD88) antibody (MyD88, D80F5 rabbit mAb, Cell Signaling Technology, Danvers, MA, USA), anti-TIR-domain-containing adapter-inducing interferon-β (TRIF) antibody (TRIF/TICAMI antibody, N8120-13810, Novus Biologicals, Centennial, CO, USA), anti-NLR family pyrin domain containing 3 (NLRP3) antibody (NLRP3 antibody [NBP2-1244], Novus Biologicals, US), anti-caspase-1 (p20) antibody (caspase-1 [p20] antibody, AG-20B-0042-C100, AdipoGen, San Diego, USA), FGF21 antibody (FGF21 antibody [EPR8314(2)], Abcam, Cambridge, MA, USA) and secondary antibodies (horser adish peroxidase [HRP] donkey anti-rabbit immunoglobulin G [IgG] antibody 406401; HRP goat anti-mouse IgG antibody 405306, Biolegend, San Diego, CA, USA). The membranes were washed and treated an enhanced chemiluminescence detection system (PerkinElmer, Waltham, MA, USA) to develop the immune complexes, and the bands were quantified through analysis with the BioSpectrum AC image system, UVP Visionwork LS software (v. 8), and Image-Pro Plus 4.5 (Media Cybernetic, Rockville, MD, USA). Equal loading of the total protein was controlled with a commercially available antibody against glyceraldehyde 3-phosphate dehydrogenase (GAPDH, BioLegend, San Diego, CA, USA) or α-Tubulin (Abcam, Cambridge, UK). The results are expressed as the relative ratio of the target protein level to the control protein level.

### 2.6. Fecal Analysis

DNA was extracted from randomly selected fresh fecal samples with a QIAamp DNA Stool Mini Kit (Qiagen, ST, USA) according to Godon’s method [17]. After extraction, V3-V4 regions of 16S rDNA were amplified through Illumina HiSeq Sequencing to obtain raw data. UCHIME was used to generate sequences as clusters [18] and conduct filtering from the effective tags (>97% sequence identity). Abundance information was selected from organization taxonomic units (OTU) to ensure a minimum number of sequences per sample and avoid sampling depth bias. Alpha diversity was calculated to evaluate the richness, evenness, and diversity of fecal microbiota. Variations in the species complexity of samples were examined according to the beta diversity. Principal component analysis (PCA) was performed to compress and classify data, and group differences in microbial community composition were assessed using permutational multivariate analysis of variance (PERMANOVA). Differences between groups were evaluated according to the relative abundances of microbial species displayed at the phylum, class, order, family, genus, and species levels. Differential microbial abundance between groups was analyzed using Metastats, and *p*-values were adjusted for multiple comparisons using the false discovery rate (FDR) method to control for type I errors [19]. The Firmicutes-to-Bacteroidetes (F-B) ratio was also calculated. The taxonomic classification of the sequences per sample was annotated according to the Ribosomal Database Project classifier (version 2.11). The linear discriminant analysis effect size method was used to determine major taxa and biomarkers in the three groups based on relative abundances with a non-parametric factorial Kruskal–Wallis rank-sum test [20].

### 2.7. Statistical Analysis

The SAS program (v. 9.4; Cary, NC, USA) was used for statistical analysis, with values expressed as the mean and standard error of the mean (SEM). This experiment was not performed blinded. One-way analysis of variance (ANOVA) and Tukey’s multiple range test were performed to compare data among groups following the experiment. Spearman’s correlation analysis was performed to analyze the correlation between two variables. A *p* value < 0.05 was considered significant.

Sample size estimation was conducted using G*Power 3.1.9.7 for one-way ANOVA with three groups (α = 0.05, power = 0.80) and regarding hepatic TG level as the main parameter, indicating a minimum of 6 animals per group to detect an effect size of Cohen’s f = 0.75. To ensure sufficient power and account for experimental variability, we used 9 animals per group (total n = 27), which allows for the detection of effect sizes as small as f = 0.637 (medium to large range).

## 3. Results

### 3.1. Energy Intake and Body and Tissue Weight

Mice were pair-fed isocaloric liquid diets during the experiment. No differences in body weight and retroperitoneal and epididymal WAT weight were noted between groups. Liver and mesenteric adipose tissue weights were significantly lower in the KD and KS groups than in the C group, but no difference was observed between the KD and KS groups (Table 1).

### 3.2. Effects of Ketogenic Diets on Carbohydrate and Lipid Metabolism

**Figure 1:**
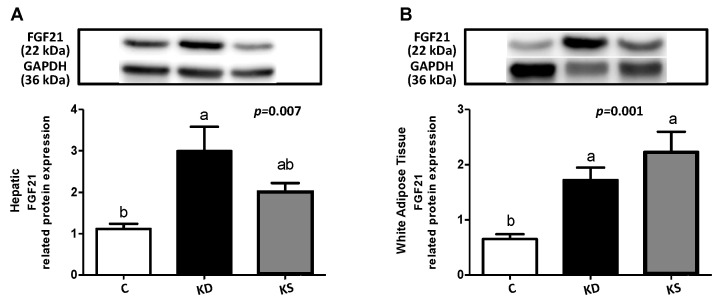
(**A**) Hepatic and (**B**) WAT FGF21 relative protein expression in mice. Values represent means ± SEM; n = 6–9 per group. One-way ANOVA and subsequent Tukey’s multiple range tests were performed. Lower-case letters denote significant differences between groups in each panel. FGF21, fibroblast growth factor 21; C, control diet; KD, ketogenic diet; KS, ketogenic diet with soy protein.

Both the KD and KS groups were administered ketogenic diets and exhibited lower fasting plasma glucose and insulin levels than the C group, with no difference observed between the KD and KS groups (Table 2). Only the KD group had a higher plasma β-hydroxybutyrate concentration than the C group. No significant difference in FFA (*p* = 0.3645) and TG (*p* = 0.2168) concentrations was observed between the three groups. The KD group exhibited higher TC and HDL-C levels than the C group, and the KS group exhibited higher TC, HDL-C, LDL-C, and LDL/HDL levels than both the KD and C groups. In the hepatic index analysis, AST and ALT were generally lower in the KD and KS groups than in the C group, but all values fell within the normal range for mice [21]. FGF21 is implicated in the regulation of carbohydrate and lipid metabolism; accordingly, significantly higher circulating levels of FGF21 were observed in the KD and KS groups than in the C group. Moreover, hepatic FGF21 protein expression was higher in the KD group than in the C group, and WAT FGF21 protein expression was higher in both the KD and KS groups than in the C group. These findings suggest that FGF21 may regulate carbohydrate and lipid metabolism in the liver and WAT (Figure 1).

**Figure 2:**
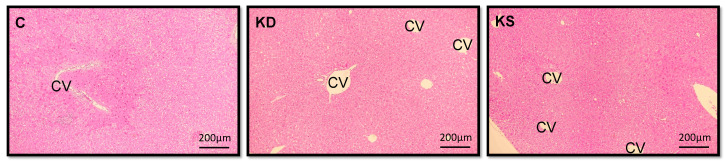
Histopathological analysis of liver sections (hematoxylin and eosin staining, original magnification × 100). C, control diet; KD, ketogenic diet; KS, ketogenic diet with soy protein. CV, central vein.

Tissue sections were taken from the biggest live lobe of each mouse for histopathological examination, with no differences in morphological characteristics observed between the three groups (Figure 2). Likewise, no significant differences in hepatic FFA (*p* = 0.2046), TG (*p* = 0.2412), or TC (*p* = 0.5931) levels were observed between groups (Table 2).

**Figure 3:**
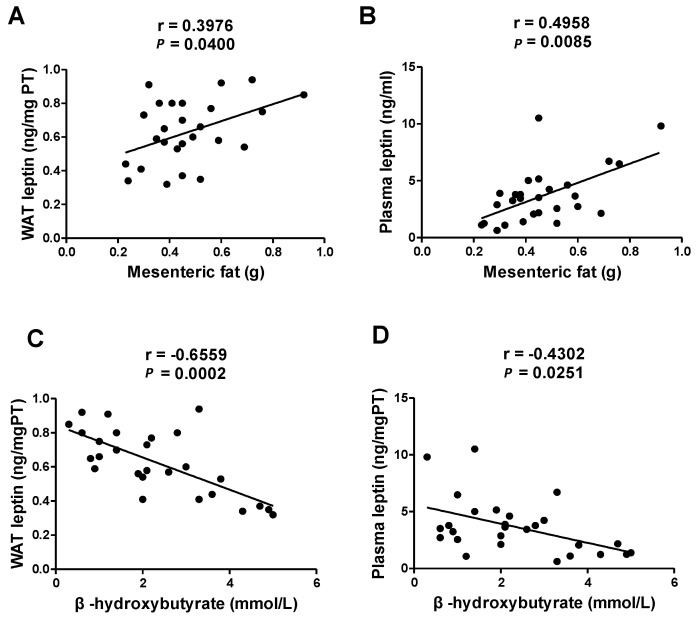
Linear regression plot of (**A**) WAT leptin levels correlated with mesenteric fat weight (**B**) plasma leptin levels correlated with mesenteric fat weight, (**C**) WAT leptin levels correlated with plasma β-hydroxybutyrate levels, and (**D**) plasma leptin levels correlated with plasma β-hydroxybutyrate levels. *p* < 0.05 and Spearman’s correlation coefficient (r) > 0.4.

We also measured adipokine concentrations. Although no significant differences in leptin or adiponectin levels in plasma and WAT were observed between groups (Table 3), leptin concentrations in plasma and WAT were positively associated with mesenteric WAT weight and plasma β-hydroxybutyrate levels (Figure 3).

### 3.3. Effects of Ketogenic Diets on Inflammatory Responses

**Figure 4:**
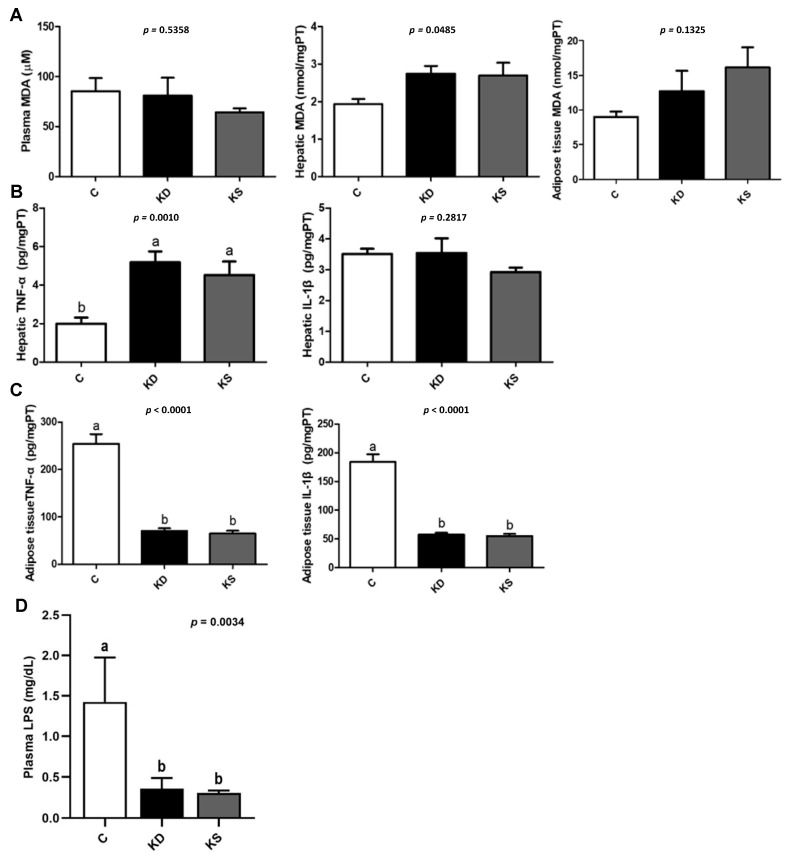
(**A**) Plasma, hepatic, and WAT MDA. (**B**) Hepatic TNF-α and IL-1β. (**C**) WAT TNF-α and IL-1β. (**D**) Plasma LPS concentrations. Values represent means ± SEM and n = 6–9. One-way ANOVA followed by Tukey’s multiple range test analysis, with lower-case letters denoting significantly different data groups in each panel. MDA, malondialdehyde; TNF-α, tumor necrosis factor alpha; IL-1β, interleukin 1beta; LPS, lipopolysaccharides. C, control diet; KD, ketogenic diet; KS, ketogenic + soy protein diet.

**Figure 5:**
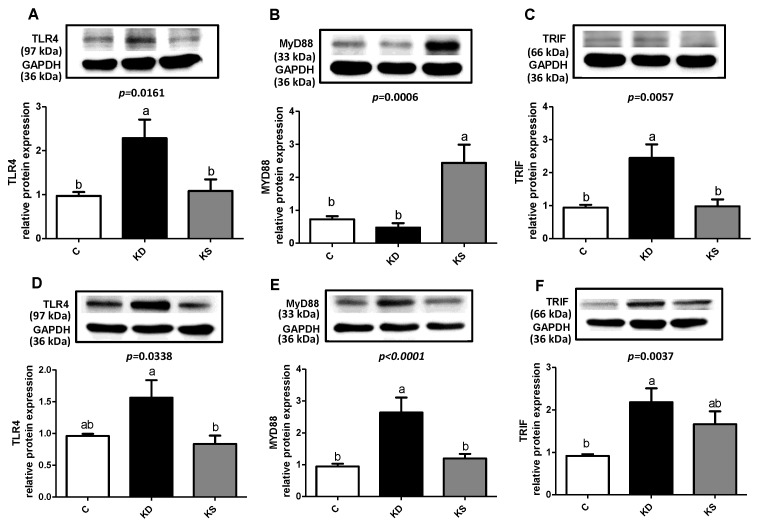
Hepatic (**A**) TLR4, (**B**) MyD88, (**C**) TRIF; and WAT (**D**), TLR4 (**E**), MyD88 (**F**) TRIF relative protein expression in mice. Values represent means ± SEM and n = 6–9. One-way ANOVA followed by Tukey’s multiple range test analysis, with lower-case letters denoting significantly different data groups in each panel. TLR4, toll-like receptor 4; MyD88, myeloid differentiation primary response 88; TRIF, TIR-domain-containing adapter-inducing interferon-β; C, control diet; KD, ketogenic diet; KS, ketogenic + soy protein diet.

**Figure 6:**
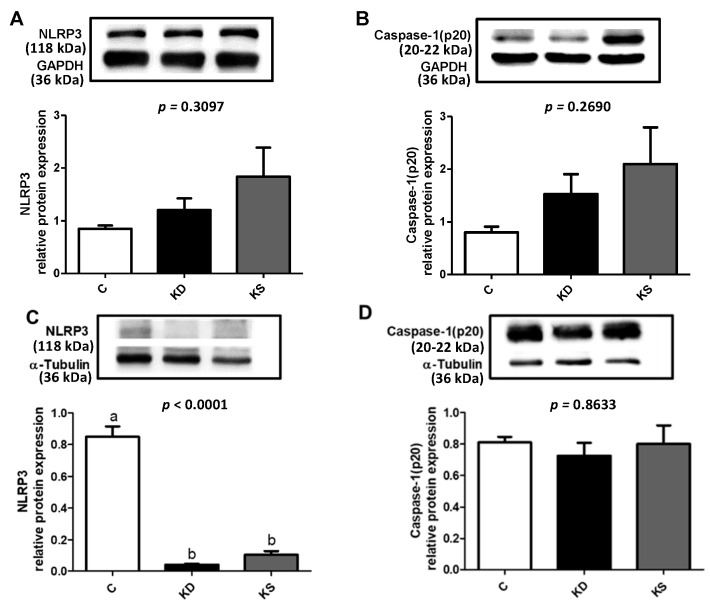
Hepatic (**A**) NLRP3 (**B**)Caspase-1 (p20); and WAT (**C**), NLRP3 (**D**) Caspase-1 (p20) relative protein expression in mice. Values represent means ± SEM and n = 6–9. One-way ANOVA followed by Tukey’s multiple range test analysis, with lower-case letters denoting significantly different data groups in each panel. NLRP3, NLR family pyrin domain containing 3; C, control diet; KD, ketogenic diet; KS, ketogenic + soy protein diet.

No significant differences in the concentration of MDA, a marker of lipid peroxidation, were observed in plasma, liver, or WAT between the three groups (Figure 4A). The analysis of proinflammatory cytokines in the liver and WAT revealed that both the KD and KS groups had higher hepatic TNF-α concentrations and lower WAT TNF-α and IL-1β concentrations than the C group (Figure 4B,C). We then analyzed the expression of related proteins to clarify possible mechanisms. Although plasma endotoxin levels were higher in the C group than in the other two groups (Figure 4D), the KD group exhibited significantly higher hepatic TLR4 and TRIF expression than the C and KS groups. TLR4, MYD88 and TRIF expression in WAT exhibited a similar pattern (Figure 5). However, contrasting findings were obtained from the analysis of pro-inflammatory cytokine concentration in WAT. Findings in the literature have indicated that β-hydroxybutyrate affects NLRP3 inflammasome expression [22]. We therefore analyzed inflammasome-related protein expression (Figure 6). Although the KS group exhibited the highest hepatic NLRP3 expression, no difference in p20 concentration was observed between the three groups (*p* = 0.2351). Conversely, KD and KS exhibited lower NLRP3 expression in WAT, and the protein analysis of p20 showed no statistically significant difference among groups.

### 3.4. Effects of Ketogenic Diets on Gut Microbiota

**Figure 7:**
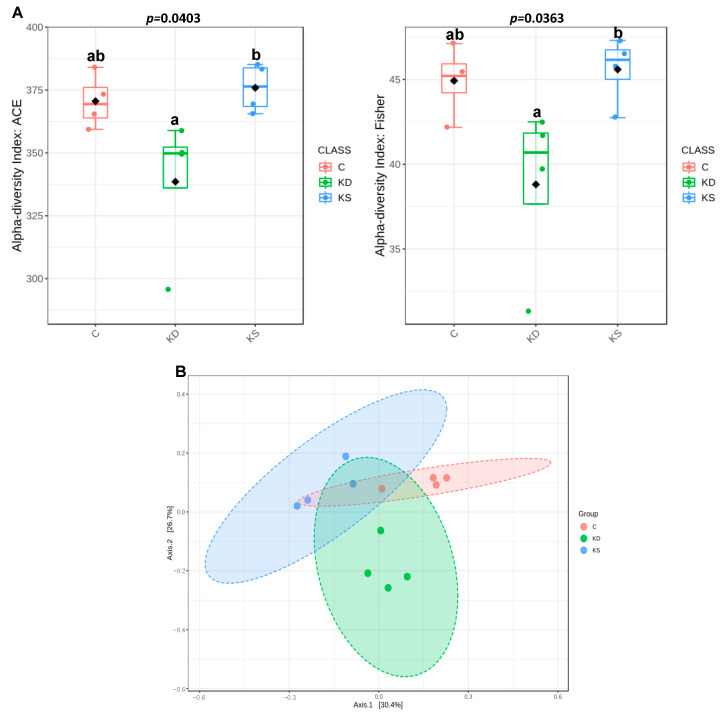
PCA analysis showing differences in terms of species in fecal samples. (**A**) Alpha diversity is shown on the box plot, and (**B**) beta diversity on the correction matrix. Lower-case letters denote significant differences between groups in each panel. C, control diet; KD, ketogenic diet; KS, ketogenic + soy protein diet.

**Figure 8:**
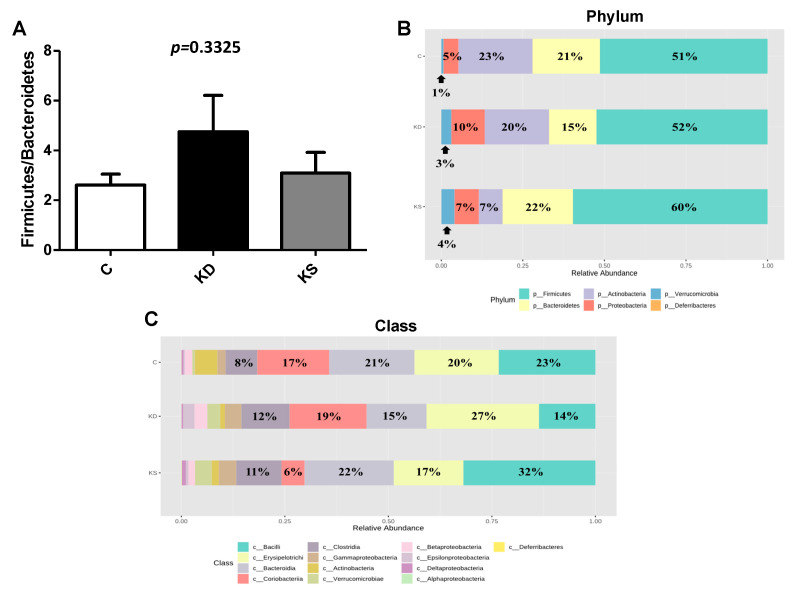

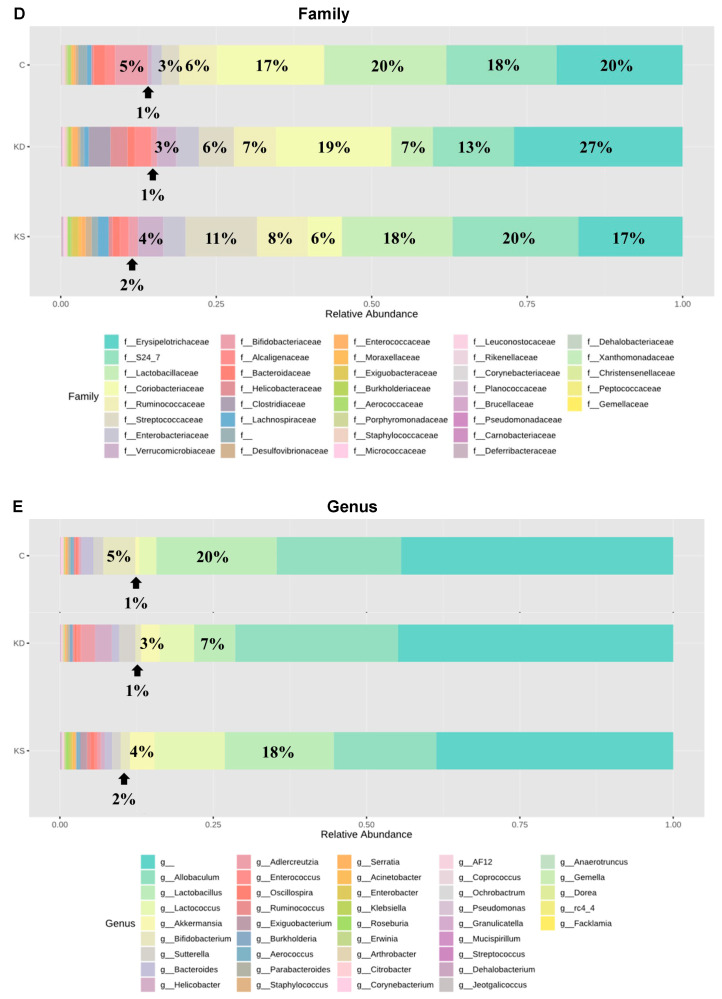
Relative abundance of bacterial communities in mice under different diets (*n* = 4). (**A**) Firmicutes/Bacteroidetes ratio; (**B**) top 10 relative abundance of microbial species at the phylum levels in the feces of mice; (**C**) the class analysis; (**D**) the family analysis; (**E**) the genus analysis. C, control diet; KD, ketogenic diet; KS, ketogenic + soy protein diet.

Dietary patterns affect gut microbiota composition and are thus of biophysiological consequences. We therefore analyzed the species richness of gut microbiota through next-generation 16S rDNA gene sequencing of randomly selected fresh fecal samples from four mice in each group. Microbiome analysis was performed on only four animals per group. According to the abundance-based coverage estimator and Fisher index, α-diversity was significantly higher in the KS group than in the KD group, whereas no significant difference was observed between the C group and either the KD or KS group (Figure 7A). PCA was performed to analyze beta diversity, revealing differences in microbial composition between the three groups (Figure 7B). There was no significant difference in the F-B ratio among the C, KD, and KS groups (Figure 8A).

A further analysis of the microbial composition of each sample at different taxonomic levels revealed that mice in the KD and KS groups had more abundant Firmicutes (phylum), Verrucomicrobia (phylum), Clostridia (class), and Akkermansia (genus) compared with the C group. Between the two groups administered ketogenic diets with different protein sources, the KS group exhibited a greater relative abundance of Firmicutes (phylum), Bacteroidetes (phylum), Lactobacillaceae (family), and Lactobacillus (genus) than the KD group. Additionally, the KD group exhibited higher Coriobacteriaceae (family) composition than the C and KS groups (Figure 8B–E). A negative correlation was observed between the concentration of plasma lipopolysaccharides (LPS) and Akkermansia muciniphila abundance (r = −0.8264, *p* = 0.0114), and the concentration of plasma β-hydroxybutyrate was negatively correlated with Bifidobacteriaceae abundance (r = −0.6025, *p* = 0.0382).

## 4. Discussion

As obesity has become a pressing public health concern, ketogenic diets have gained considerable attention for their potential to facilitate rapid weight loss [3]. However, differences in experimental nutritional conditions, such as very low or unrestricted calorie intake, fat ratios, and carbohydrate ratios, may result in a diverse range of outcomes, and the advantages and disadvantages of ketogenic diets remain under debate [5]. To eliminate potential inconsistencies in dietary conditions, mice were administered isocaloric and isoprotein liquid diets. We also explored the potential effects of soy protein sources in a ketogenic diet. Previous studies demonstrate that various bioactive components within soy protein, including isoflavones and saponins, play a role in lipid metabolism regulation [23,24]. Furthermore, soy protein is primarily composed of glycinin and β-conglycinin. Our previous study has also shown that β-conglycinin can improve insulin resistance and slow the progression of renal function deterioration in DM rats [24]. Following the experiment, no significant differences in body weight were observed between all groups, possibly because we implemented pair-feeding throughout the experiment. However, mesenteric WAT was significantly lower in both the KD and KS groups (those administered ketogenic diets) than in the C group. In a previous experiment with obese mice, the administration of a ketogenic diet for 8 weeks (94% fat, 6% protein, <1% carbohydrate) reduced both body weight and body fat; however, these reductions did not differ significantly from those observed in mice fed a standard chow diet [25]. In another study, body weight and fat mass were significantly higher in mice fed a ketogenic diet with normal protein content (83.9% fat, 16.1% protein, 0% carbohydrate) compared with those on a standard chow diet [26]. These results suggested that dietary macronutrient ratio and appetite may affect body weight and composition. An extended intervention period may allow researchers to observe the effect of ketogenic diets on abdominal fat accumulation.

In the present study, mice fed a ketogenic diet had higher blood ketone concentrations and lower blood glucose and insulin concentrations than those administered a liquid diet, but ketogenic diets also caused dyslipidemia. Both circulating and hepatic FGF21 levels were significantly elevated in the ketogenic diet groups. FGF21 is primarily secreted by the liver and can promote lipolysis and energy production from adipose tissue, potentially explaining the lower WAT mass observed in the groups fed a ketogenic diet. Previous studies have reported that ketogenic diets upregulate hepatic FGF21 gene expression, potentially facilitating metabolic adaptation from a high-fat to ketogenic diet [25]. Previous studies have reported that ketogenic diets cause dyslipidemia and hepatic steatosis in addition to weight loss [4,5]. A study comparing the effects of a low-carbohydrate diet and a ketogenic diet in type 2 diabetic mice revealed that although both diets lowered blood glucose and improved glucose tolerance and insulin sensitivity, the ketogenic diet reduced glycogen storage and inhibited AMP-activated protein kinase phosphorylation, thereby increasing gluconeogenesis. Furthermore, the ketogenic diet was associated with greater hepatic lipid accumulation than the low-carbohydrate diet [27]. In a 6-week clinical trial, both ketogenic and nonketogenic low-carbohydrate diets had similar effects on body weight and insulin resistance, but the ketogenic diets caused several adverse metabolic effects [28]. However, in our study, pathological and hepatic analysis revealed no pronounced steatosis.

Between the two groups fed ketogenic diets with different protein sources, the KS group had higher blood cholesterol levels than the KD group. Additionally, although the KS group had generally higher blood ketone levels than the C group, no significant difference was observed between the KS and KD or C groups. Ketone body metabolism may affect cholesterol homeostasis [29]. Bielohuby et al. compared low-carbohydrate diets with diets with different fat and protein ratios, observing increased serum β-hydroxybutyrate levels when fat and protein accounted for 92.8% and 5.5% or 86.3% and 11.8% of dietary calories, respectively. However, this increase was significantly greater for the diet with higher fat content, which was also associated with higher expression of 3-hydroxy-3-methylglutaryl coenzyme A (HMG-CoA) lyase, a key enzyme in ketone body production [30]. Besides, high-fat diets may activate hepatic HMG-CoA reductase expression, thereby increasing cholesterol biosynthesis [31]. The indigestible portion of soy protein may function as dietary fiber, improving blood lipids and increasing intestinal lipid excretion [32]. These observations suggested that soy protein sources may reduce lipid absorption and affect the dietary caloric ratio of macronutrients in extremely high-fat ketogenic diets, in turn affecting the efficiency of ketone production in fatty acid metabolism and increasing cholesterol synthesis. Further studies are needed to clarify the relationship between ketone bodies and cholesterol metabolism.

Prior research has also demonstrated that an increase in WAT promotes the production and release of the adipokine leptin, which reduces appetite and increases energy expenditure [33]. Although we observed no differences in leptin concentrations among the three groups, mice fed a ketogenic diet had lighter mesenteric WAT and higher blood ketone concentrations. Leptin concentrations in both plasma and WAT were positively correlated with mesenteric fat weight and negatively correlated with ketone body levels. Future studies involving longer-term ketogenic diet interventions or animal models with pre-existing metabolic conditions can allow researchers to observe changes in the adipokine balance and its regulatory effects on proteins related to liver lipid metabolism. However, findings from other studies involving ketogenic diets have been inconsistent. For instance, Luukkonen et al. reported that in obese human subjects, ketogenic diet intake led to increased circulating ketone bodies but decreased leptin levels [34]. In contrast, Liu et al. found that leptin levels were significantly elevated in rats fed a ketogenic diet [35]. These discrepancies suggest that the relationship between ketone bodies and adipokines such as leptin may be context-dependent, and further studies are needed to elucidate the underlying regulatory mechanisms and causative relationships.

Hepatic inflammation can result from various mechanisms, including oxidative stress [36], ferroptosis [37], and the activation of inflammasomes via DAMPs, particularly through the NLRP3 and TLR4 pathways [38]. We measured the levels of hepatic oxidative stress (MDA levels), inflammasome, and the TLR4 signaling pathway. β-hydroxybutyrate may exert antioxidant effects by activating nuclear factor erythroid 2-related factor 2 and regulating the expression of downstream antioxidant enzymes. The potential anti-inflammatory effects of β-hydroxybutyrate include inhibiting LPS-induced inflammatory responses in macrophages, reducing NLRP3 inflammasome activity, and reducing IL-1β and IL-18 excretion [22,39]. Although the KD and KS groups had higher hepatic MDA levels than the C group, this difference was nonsignificant. Hepatic proinflammatory cytokine TNF-α concentrations were higher in the KD and KS groups compared with the C group, but TNF-α and IL-1β concentrations in WAT were lower in the KD and KS groups than in the C group. Likewise, Asrih et al. observed increased hepatic proinflammatory cytokine expression but decreased proinflammatory cytokine expression in epididymal WAT in C57BL/6J mice fed a ketogenic diet for 4 weeks [40]. Although a high-fat diet may be associated with endotoxemia, ketogenic diets have improved inflammatory responses in mice with endotoxemia [41]. In addition, a review article on FGF21 has highlighted its beneficial role in regulating carbohydrate and fatty acid metabolism in white adipose tissue [42]. In our study, FGF21 levels in adipose tissue were significantly higher in both the KD and KS groups compared to the control group, which may have contributed to improved energy metabolism in adipose tissue and, consequently, reduced inflammatory responses. However, although hepatic FGF21 expression was highest in the KD group, the hepatic proinflammatory cytokine levels did not show a consistent pattern.

Circulating LPS concentration was lower in the KD and KS groups compared with the C group. A further examination of protein expressions related to the TLR4 signaling pathway in the liver and adipose tissue revealed higher hepatic TLR4 and TRIF protein expression in the KD group than in the C and KS groups. Hepatic MyD88 protein expression was higher in the KS group than in the C group. High-fat diets may activate the TLR2/4 signaling pathway. Both TLR2 and TLR4 are upstream factors related to MyD88 that can also promote NF-κB activation, increasing the release of proinflammatory cytokines [43]. Cohen-Sfady et al. suggested that inflammatory responses promote the expression of heat shock proteins, which may activate B cells through the TLR4/MyD88 signaling pathway, further promoting inflammation [44]. However, higher expression of heat shock proteins has been observed in the adipose tissue of ketotic calves, possibly indicating a compensatory adaptive response [45]. Therefore, although we observed lower circulating LPS levels in the KD and KS groups, heat shock proteins or other metabolic abnormalities may have activated TLR4 and downstream signaling pathways, increasing the release of proinflammatory cytokines in the liver. Recent studies have indicated that β-hydroxybutyrate may regulate the NLRP3 signaling pathway in mice to inhibit the release of the proinflammatory cytokine IL-1β [22,46]. In our study, both the KD and KS groups had higher blood β-hydroxybutyrate concentrations than the C group. However, no significant difference in hepatic NLRP3 protein expression was observed between the three groups. Although the exact mechanism through which β-hydroxybutyrate affects NLRP3 function in different tissues remains uncertain, it should be considered in future investigations into the inflammatory effects of ketogenic diets in various tissues.

Diet may regulate metabolism and biophysiological functions through the gut–liver–lipid axis. A recent study reported that high-fat diets may affect the gut microbiota and increase bacterial translocation, increasing the concentration of circulating endotoxins and exacerbating tissue inflammation and damage [47]. Another study reported that ketogenic diets affect microorganism diversity and gut microbiota balance, such as by changing the F-B ratio [48]. In our experiment, mice fed a ketogenic diet had lower gut microbial species richness than those fed a control diet. Among the ketogenic diet groups, species richness was higher in the KS group than in the KD group. This result is consistent with Watanabe et al.’s finding that a high-fat diet featuring soy protein sources altered gut microbiota composition [49]. Zhao et al. reported that a high-fat diet may increase the abundance of Coriobacteriaceae [50], which may be involved in bile acid metabolism and cholesterol absorption, thereby affecting blood cholesterol levels. Although both ketogenic diet groups had higher blood cholesterol concentrations than the C group, the KS group had the lowest abundance of Coriobacteriaceae among all groups. The components in soy protein may have inhibited Coriobacteriaceae abundance [51], suggesting that the higher blood cholesterol levels caused by ketogenic diets in this study cannot be explained by this particular bacterial group.

The abundance of Verrucomicrobia and *Akkermansia muciniphila* is reportedly related to the regulation of intestinal barrier integrity. Nagpal et al. reported that a 6-week intervention comprising a modified Mediterranean ketogenic diet increased the relative abundance of Verrucomicrobiaceae and decreased that of Bifidobacteriaceae compared with a low-fat diet, although it did not significantly affect the abundance of Firmicutes and Bacteroides [52]. Similarly, we observed a higher relative abundance of *A*. *muciniphila* and lower relative abundances of Bifidobacteria and Lactobacillus in the KD group than in the C group. Compared with the KD group, the KS group exhibited higher relative abundances of Verrucomicrobia, Bifidobacteria, and Lactobacillus, and *A. muciniphila* was negatively correlated with circulating LPS concentrations. However, higher hepatic proinflammatory cytokine levels were observed in both the KD and KS groups compared with the C group, suggesting that ketogenic diets did not increase liver inflammatory damage through the gut–liver axis in our experiment.

Despite these findings, this study still has some limitations. While liver histology was evaluated blindly by an independent pathologist, other procedures were not blinded due to limited resources, which still may possibly introduce bias. Only male mice were used, limiting generalizability across sexes. The study duration was relatively short, and the limited sample size for microbiome analysis may have reduced statistical power. Long-term mixed-effects models may be considered for analyzing data such as longitudinal body weight data to better account for within-subject variability. Additionally, while Tukey’s test was used in this study to control type I error, more conservative corrections such as Bonferroni may be applied in future work to enhance statistical rigor. In addition, the mouse model in the present study was not disease-induced, and future studies may further investigate the effects of ketogenic diets and soy protein substitution in pathological conditions to improve clinical relevance. It would also be beneficial to incorporate additional physiological assessments such as energy expenditure, physical activity, glucose tolerance tests or assessments of gut permeability and lipid metabolism to provide a more complete understanding of metabolic responses and strengthen future findings. Overall, these findings provide preliminary insights, and further research is needed to clarify the long-term metabolic effects of ketogenic and soy protein-based diets across different sexes and disease states.

## 5. Conclusions

The gradual transition toward a 5% carbohydrate of total energy ketogenic diet under isocaloric intake conditions did not cause greater weight loss compared with a balanced diet in normal mice. Ketogenic diets reduced mesenteric WAT weight, blood glucose, but caused dyslipidemia and increased hepatic proinflammatory cytokine concentrations. Additionally, the consumption of soy rather than animal protein may cause greater dyslipidemia, whereas no differences were observed in WAT weight and inflammation compared with the consumption of animal protein in a ketogenic diet. However, the soy protein-based ketogenic diet appeared to mitigate the reduction in gut microbiota diversity typically observed with ketogenic diets, and its effects on physiological regulation merit further exploration.

Findings regarding the effects of ketogenic diets on tissue inflammatory responses remain inconsistent. We controlled for isocaloric intake and observed that ketogenic diets had different effects on the liver and adipose tissues compared with a control diet and that plant-based protein sources in ketogenic diets caused dyslipidemia. Additional research is necessary to clarify the metabolic alteration mechanisms in longer-term or disease-state animal models and to identify the specific bioactive components responsible for the observed effects, thereby providing a stronger reference for potential clinical applications.

## Figures and Tables

**Table 1:** Body, liver, and adipose tissue weights of mice at the end of the experiment.

	C	KD	KS
Body weight (g)			
Initial	21.3 ± 0.2	21.2 ± 0.3	21.3 ± 0.3
Final	32.1 ± 0.8	29.9 ± 0.7	32.3 ± 1.0
Liver weight (g)	1.5 ± 0.1 ^a^	1.1 ± 0.1 ^b^	1.2 ± 0.0 ^b^
Mesenteric fat (g)	0.6 ± 0.1 ^a^	0.4 ± 0.0 ^b^	0.4 ± 0.0 ^b^
Retroperitoneal fat (g)	0.4 ± 0.0	0.4 ± 0.0	0.5 ± 0.1
Epididymal fat (g)	1.0 ± 0.1	0.9 ± 0.1	1.2 ± 0.1

Values represent means ± SEM; *n* = 9 per group. One-way ANOVA and subsequent Tukey’s multiple range tests were performed. Lower-case letters denote significant differences between groups in each panel. C, control diet; KD, ketogenic diet; KS, ketogenic diet with soy protein.

**Table 2:** Biochemical parameters of mice at the end of the experiment.

	C	KD	KS
*Blood:*			
glucose (mg/dL)	230.4 ± 14.1 ^a^	167.9 ± 7.9 ^b^	190.9 ± 10.0 ^b^
β-hydroxybutyrate (mmol/L)	1.2 ± 0.2 ^b^	3.2 ± 0.4 ^a^	2.6 ± 0.5 ^ab^
insulin (µg/L)	1.8 ± 0.4 ^a^	0.4 ± 0.0 ^b^	0.4 ± 0.0 ^b^
FFA (µmol/mL)	260.4 ± 27.7	365.4 ± 54.4	355.3 ± 68.7
TG (mg/dL)	59.7 ± 6.1	77.0 ± 11.3	57.8 ± 6.4
TC (mg/dL)	74.9 ± 5.0 ^c^	92.2 ± 2.7 ^b^	111.4 ± 3.2 ^a^
HDL-C (mg/dL)	67.9 ± 4.7 ^c^	85.7 ± 3.3 ^b^	103.7 ± 3.0 ^a^
LDL-C(mg/dL)	5.9 ± 0.6 ^b^	7.3 ± 0.3 ^b^	15.4 ± 1.0 ^a^
LDL-C/HDL-C (%)	8.6 ± 0.4 ^b^	8.6 ± 0.3 ^b^	14.8 ± 0.7 ^a^
AST (U/L)	73.9 ± 7.6 ^a^	49.9 ± 2.5 ^b^	43.4 ± 2.2 ^b^
ALT (U/L)	56.0 ± 10.3 ^a^	27.8 ± 2.0 ^b^	27.9 ± 1.4 ^b^
FGF21 (µg/mL)	3.2 ± 0.4 ^b^	5.5 ± 0.1 ^a^	5.9 ± 0.1 ^a^
*Liver:*			
FFA (mmol/g liver)	2.3 ± 1.2	3.4 ± 0.4	4.4 ± 0.6
TG (μmol/g liver)	9.8 ± 1.8	9.1 ± 1.2	6.7 ± 0.7
TC (μmol/g liver)	13.9 ± 2.1	14.0 ± 2.0	11.6 ± 1.3

Values represent mean ± SEM; *n* = 6–9 per group. One-way ANOVA and subsequent Tukey’s multiple range tests were performed. Lower-case letters denote significant differences between groups in each panel. C, control diet; KD, ketogenic diet; KS, ketogenic diet with soy protein; FFA, free fatty acids; TG, triglycerides; TC, total cholesterol; HDL-C, high-density lipoprotein cholesterol; LDL-C, low-density lipoprotein cholesterol; AST, aspartate aminotransferase; ALT, alanine aminotransferase.

**Table 3:** Plasma and white adipose tissue adipokines in mice.

	C	KD	KS
*Plasma*			
Adiponectin (ng/mL)	506.6 ± 16.7	492.9 ± 12.8	486.9 ± 14.7
Leptin (ng/mL)	4.1 ± 0.9	2.5 ± 0.5	4.5 ± 0.9
A/L (%)	178.1 ± 38.8	307.8 ± 76.0	156.0 ± 37.7
*WAT*			
Adiponectin (ng/mg PT)	20.6 ± 0.9	20.6 ± 1.1	17.8 ± 0.4
Leptin (ng/mg PT)	0.8 ± 0.1	0.6 ± 0.1	0.6 ± 0.1
A/L (%)	28.4 ± 2.4 ^b^	40.4 ± 4.3 ^a^	33.8 ± 3.2 ^ab^

Values represent mean ± SEM; *n* = 9 per group. One-way ANOVA and subsequent Tukey’s multiple range tests were performed. Lower-case letters denote significant differences between groups in each panel. C, control diet; KD, ketogenic diet; KS, ketogenic diet with soy protein; WAT, white adipose tissue; A/L = adiponectin/leptin.

## Data Availability

The original contributions presented in this study are included in the article and Supplementary Materials. Further inquiries can be directed to the corresponding author.

## Supplementary Materials

The following supporting information can be downloaded at https://www.mdpi.com/article/10.3390/nu17152428/s1, Table S1: Composition of experimental liquid diets (g/L diet); Figure S1: The LEfSe method identifies the most differentially enriched taxa in each group.
