# Supplementary material for: Effects of Soy Protein on Liver and Adipose Tissue Inflammation and Gut Microbiota in Mice Fed with Ketogenic Diets
Source: Nutrients. 2025 Jul 25;17(15):2428. doi: 10.3390/nu17152428 (PMC12348106; doi:10.3390/nu17152428)
Supplement: Supplementary file 1 [file nutrients-17-02428-s001.zip › nutrients-3742896-supplementary.pdf]

**Table S1:** Composition of experimental liquid diets (g/L diet)

| Ingredient | C | LC | KD | KS |
|---|---|---|---|---|
| Casein | 28 | 28 | 28 | 0 |
| Soy protein | 0 | 0 | 0 | 28 |
| Methionine | 0.3 | 0.3 | 0.3 | 0.3 |
| L-cystine | 0.5 | 0.5 | 0.5 | 0.5 |
| Dextrin | 192 | 22 | 12 | 12 |
| Sucrose | 0 | 23 | 0 | 0 |
| Soybean oil | 10 | 75 | 90 | 90 |
| Cellulose | 10 | 10 | 10 | 10 |
| Xanthan gum | 3 | 3 | 3 | 3 |
| Mineral | 15 | 15 | 15 | 15 |
| Vitamin | 5 | 5 | 5 | 5 |
| Choline | 0.53 | 0.53 | 0.53 | 0.53 |
| Caloric density (kcal/L) | 973.2 | 970.2 | 973.2 | 973.2 |

Casein high nitrogen, L-cysteine, methionine, choline bitartrate, cellulose, mineral mixture (AIN-93M) and vitamin mixture (AIN-93M) were acquired from ICN biochemical (Aurora, OH, USA). Soy protein was obtained from the Fuji Oil Co. Ltd. (Osaka, Japan). Soybean oil, sucrose was obtained from Taiwan Sugar Corporation (Tainan, Taiwan). Dextrin-maltose was obtained from Ingredion Incorporated (Westchester, IL, USA). Xanthan gum was purchased from Sigma (SI. Louis, MO, USA). C, control diet; LC, low-carbohydrate-high-fat diet; KD, ketogenic diet; KS, ketogenic + soy protein diet.

**A**

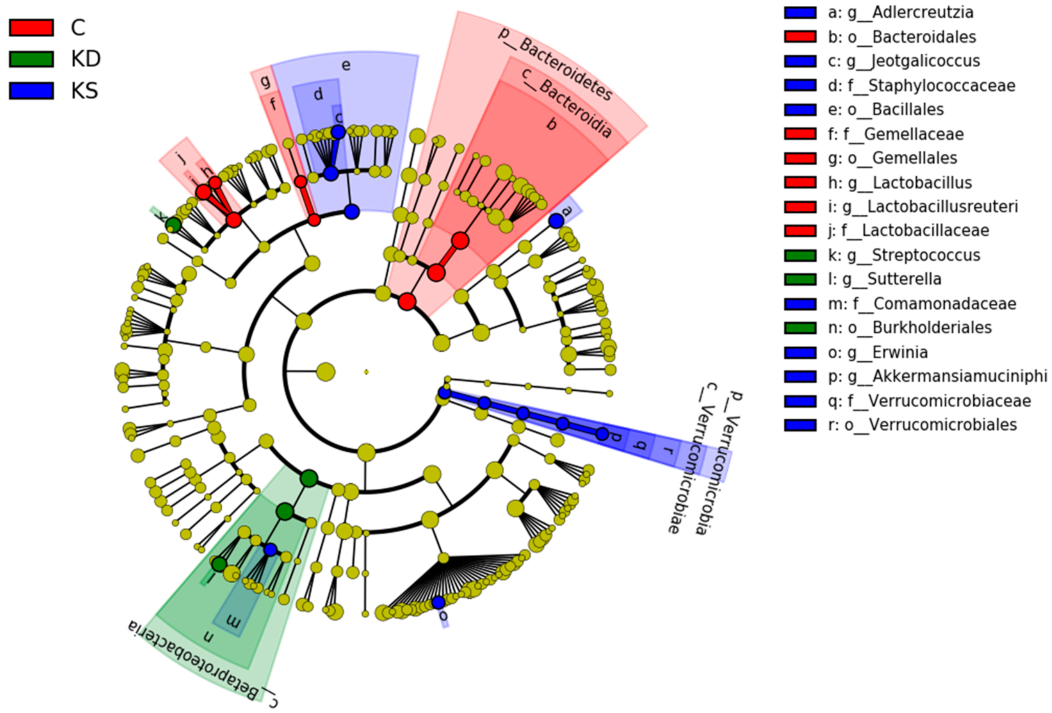

**B**

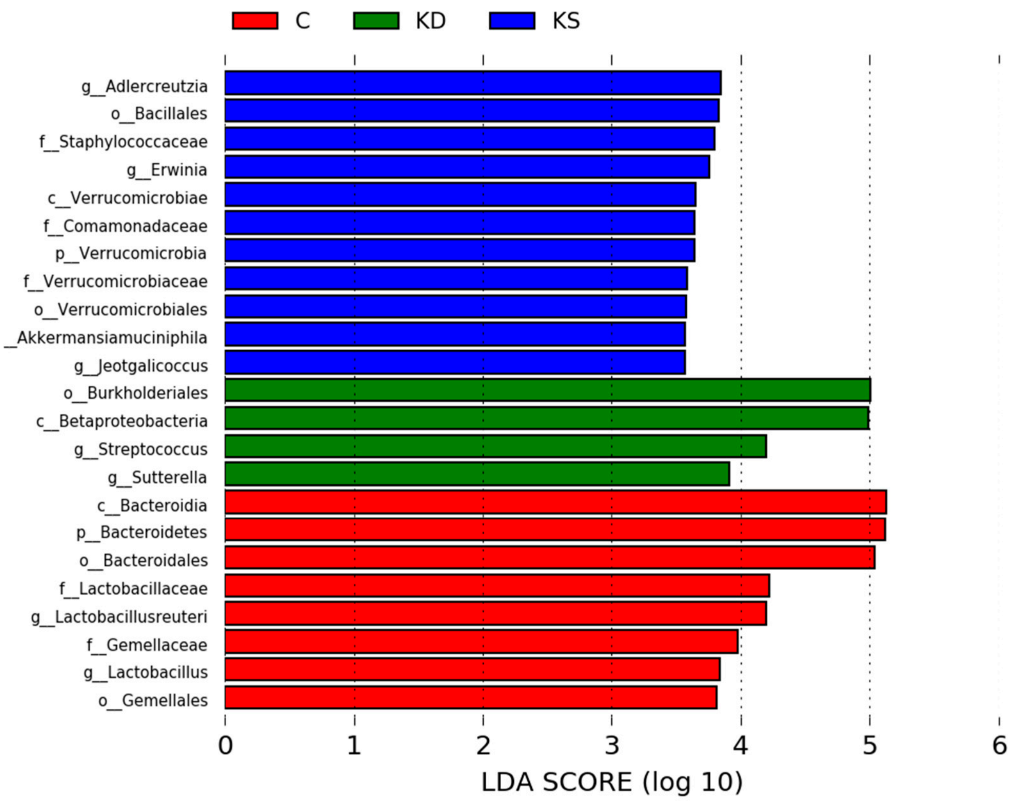


**Figure S1:** The LEfSe method identifies the most differentially enriched taxa in each group. (A) Cladogram and (B) LDA score of bacterial community among C group (red), KD group (green), and KS group (blue). C, control diet; KD, ketogenic diet; KS, ketogenic diet with soy protein.
